# Alcoholic beverages and risk of renal cell cancer

**DOI:** 10.1038/sj.bjc.6603890

**Published:** 2007-07-24

**Authors:** J P Greving, J E Lee, A Wolk, C Lukkien, P Lindblad, A Bergström

**Affiliations:** 1 Division of Nutritional Epidemiology, Institute of Environmental Medicine, Karolinska Institutet, Stockholm, Sweden; 2 Channing Laboratory, Department of Medicine, Brigham and Women's Hospital and Harvard Medical School, Boston, MA, USA; 3 Department of Urology, Sundsvall Hospital, Sundsvall, Sweden; 4 Division of Environmental Epidemiology, Institute of Environmental Medicine, Karolinska Institutet, Stockholm, Sweden

**Keywords:** alcoholic beverages, renal cell cancer, case–control study

## Abstract

Using a mailed questionnaire, we investigated the risk of renal cell cancer in relation to different types of alcoholic beverages, and to total ethanol in a large population-based case–control study among Swedish adults, including 855 cases and 1204 controls. Compared to non-drinkers, a total ethanol intake of >620 g month^−1^ was significantly related to a decreased risk of renal cell cancer (odds ratio (OR) 0.6, 95% confidence interval (CI) 0.4–0.9; *P*-value for trend=0.03). The risk decreased 30–40% with drinking more than two glasses per week of red wine (OR 0.6, 95% CI 0.4–0.9), white wine (OR 0.7, 95% CI 0.4–1.0), or strong beer (OR 0.6, 95% CI 0.4–1.0); there was a clear linear trend of decreasing risk with increasing consumption of these beverages (*P*-values for trends <0.05).

Case–control and prospective studies have shown an inverse association between alcohol intake and risk of renal cell cancer (Goodman *et al*, 1986; Asal *et al*, 1988; Wolk *et al*, 1996; Parker *et al*, 2002; Hu *et al*, 2003; Nicodemus *et al*, 2004; Mahabir *et al*, 2005; Rashidkhani *et al*, 2005; Lee *et al*, 2006, 2007), but no association (Yu *et al*, 1986; Brownson, 1988; Maclure and Willett, 1990; Talamini *et al*, 1990; Benhamou *et al*, 1993; Hiatt *et al*, 1994; Muscat *et al*, 1995; Boeing *et al*, 1997; Lindblad *et al*, 1997; Yuan *et al*, 1998; Pelucchi *et al*, 2002) or a slightly elevated risk among the highest category of beer drinkers (McLaughlin *et al*, 1984) was found in others. In studies that showed a reduced risk (Goodman *et al*, 1986; Asal *et al*, 1988; Wolk *et al*, 1996; Parker *et al*, 2002; Hu *et al*, 2003; Nicodemus *et al*, 2004; Mahabir *et al*, 2005; Rashidkhani *et al*, 2005; Lee *et al*, 2006, 2007), the association was more pronounced for wine (Asal *et al*, 1988; Rashidkhani *et al*, 2005), wine and liquor (Goodman *et al*, 1986; Wolk *et al*, 1996), beer (Parker *et al*, 2002; Lee *et al*, 2006), or beer and liquor (Mahabir *et al*, 2005). These differences may be due to small sample sizes in many studies and they require further investigation.

We investigated the association of different types of alcoholic beverages and of total alcohol (ethanol) consumption with the risk of renal cell cancer in a large population-based case–control study in Sweden.

## MATERIALS AND METHODS

We carried out a population-based case–control study of men and women aged 20–79 years without previously diagnosed renal cell cancer (ICD-9 diagnosis code 189.0), born in Sweden or any other Nordic country and resident in Sweden between 1 January 1996 and 30 June 1998 (Bergstrom *et al*, 2001). Through regional cancer registers, we identified all incident cases of renal cell cancer (*n*=1275) in five of Sweden's six hospital regions. Patients were asked to participate through their physicians. A total of 877 cases (69%) participated in the study. Non-participation was because of death (12% of identified cases), the patient being too ill or disabled (6%), or patient refusal (13%). The cancer patients were contacted at least 1 month after diagnosis and on average after 3 months. Control subjects were randomly selected from the Swedish population registry and were frequency-matched to cases by sex, age in 10-year strata, and place of residence. Of the 2046 selected control subjects, 1508 (74%) agreed to participate, with non-participation mainly because of refusal (24% of subjects). All regional ethics committees approved the study protocol.

All case and control subjects received a self-administered questionnaire on personal and medical history, as well as dietary habits and alcohol consumption 5 years before study, disregarding recent changes. We asked about the usual frequency of consumption of medium-strong beer (2.8 g ethanol per 100 g), strong beer (4.5 g ethanol per 100 g), white wine (8.9 g ethanol per 100 g), red wine (9.9 g ethanol per 100 g), strong wine (16 g ethanol per 100 g), and hard liquor (32 g ethanol per 100 g). The respondents answered in terms of number of drinks per week, month, or year, given standard portion sizes (a glass of beer=200 ml, a glass of wine=100 ml, and a glass of strong wine or hard liquor=40 ml). Light beer (1.8 g ethanol per 100 g) consumption was given in a range of nine predetermined response categories ranging from ‘never’ to ‘three times a day or more’. We converted frequency and amount of alcohol into total grams of ethanol. The questionnaire also covered education, smoking, usual adulthood weight, height, hypertension, and diabetes. If needed, we contacted subjects by telephone for missing details. Nine cases and 284 controls failed to return the mailed questionnaire and were instead interviewed only by telephone. This short telephone interview did not include the questions on alcohol consumption. Furthermore, 13 cases and 20 controls did not answer the question on alcohol consumption in the questionnaire.

Unconditional logistic regression models were used to calculate odds ratios (OR) as estimates of relative risk and 95% confidence intervals (95% CI). Data were explored in models including only sex and age (categorised as <40, 40–49, 50–59, 60–69, 70–79 years) in addition to models with sex, age and the following covariates: cigarette smoking (never smoked, smokers stratified as low (⩽16.7 pack-years) or high (>16.7 pack-years)); usual adulthood body mass index (BMI, weight height^−2^ (kg m^−2^), stratified into quartiles); years of education (<10, 10–12, >12); hypertension (yes/no); diabetes (yes/no). Subjects who had quit smoking were classified as low (67%) or high (33%), according to their pack-years consumption. The effect of different types of alcoholic beverage was examined in separate logistic regression models in which non-drinkers of the alcoholic beverage in question served as the reference category. Median values of exposure categories were entered as continuous variables into multivariate regression models to assess the significance of linear trend with increasing exposure.

## RESULTS

A total of 855 cases and 1204 controls reported their alcohol consumption and were included in the analyses. The distributions according to age, cigarette smoking, BMI, education, hypertension, and diabetes among case and control subjects are shown in Table 1. Cases and controls were broadly similar in distribution by sex (59% male cases and 61% male controls), and age (64.3 years among cases and 64.4 among controls). The prevalence of hypertension and diabetes was higher among case subjects. Control subjects had a somewhat lower BMI, but there was no major difference in the prevalence of smoking or education. Control subjects with missing alcohol consumption had a higher BMI than control subjects who were included in the analyses. No differences in characteristics were observed between cases with missing information on alcohol consumption and those included in the analyses.

Fifteen per cent of the study population (136 cases and 179 control subjects) reported not drinking alcohol (including light beer) while two-thirds drank different types of alcoholic beverage, 11% drank only beer, 3% only wine, and 4% only strong wine or hard liquor. Consumption of white wine and red wine was correlated (Spearman correlation coefficient=0.58; 95% CI 0.55–0.60). Correlation coefficient between white wine and strong beer was 0.29 (95% CI 0.25–0.33) and between red wine and strong beer was 0.27 (95% CI 0.24–0.32). Alcohol intake was not related to BMI (*r*=−0.01; 95% CI −0.05 to 0.03). Control subjects drank more alcohol than cases, especially more wine. Men consumed beer and hard liquor more often and in greater quantities than women. Smokers drank more alcohol than non-smokers among both cases and controls.

Total ethanol intake was statistically significantly associated with a decreased risk of renal cell cancer (Table 2). The multivariate OR for more than 650 g month^−1^ (approximately 21 g day^−1^) of ethanol compared to non-users of alcohol was 0.6 (95% CI 0.4–0.9). Although a statistically significant association was observed only for the >620 g month^−1^ category, there was a significant inverse trend (*P* for trend=0.03).

Odds ratios for different types of alcoholic beverage and total ethanol intake, and risk of renal cell cancer are presented in Table 3. In multivariate models including age, sex, BMI, and cigarette smoking (Model 1), consumption of more than two glasses of red wine per week was associated with a 40% (OR 0.6, 95% CI 0.4–0.9) reduction in risk compared to non-drinkers of red wine. An inverse association was also observed, albeit of borderline statistical significance, among those drinking more than two glasses per week of white wine (OR 0.7, 95% CI 0.4–1.0) or strong beer (OR 0.6, 95% CI 0.4–1.0). The risk of renal cell cancer decreased with increasing intake frequencies of white wine (*P* for trend=0.02), red wine (*P* for trend=0.01), and strong beer (*P* for trend=0.04). We found no relation between renal cell cancer risk and drinking of either light beer, medium-strong beer, strong wine, or hard liquor. Further adjustment of the multivariate models (Model 1) for education, hypertension, and diabetes did not affect the risk estimates (data not shown).

When we additionally adjusted for the other six beverages in the multivariate regression model, this mutual adjustment did not change risk estimates for each specific alcoholic beverage (Model 2), suggesting that strong beer, white wine, and red wine were each responsible for the reduction in risk of renal cell cancer.

To investigate if there is an effect modification by sex, we performed analyses of men and women separately. In the multivariate model, significant and nonsignificant inverse associations for total ethanol, strong beer, white wine, and red wine were observed in both men and women, but were less apparent in women (data not shown). Similarly, in analyses stratified by BMI (<25.0 *vs* ⩾25.0 kg m^−2^), smoking (ever/never), and hypertension (yes/no), there were no differences between the subgroups (data not shown).

## DISCUSSION

In this population-based case–control study, we observed an inverse association between moderate alcohol intake and risk of renal cell cancer. Consumption of red wine, white wine, and strong beer was associated with a lower risk. However, there were no clear associations with light and medium beer, strong wine, or hard liquor, perhaps due to chance or differences in other risk factors related to specific types of alcoholic drink. For example, the large variation in other risk factors such as smoking and occupation could explain why hard liquor was not associated with renal cell cancer risk although we controlled for known risk factors.

The major strengths of our study are its population-based design and the large number of cases. The Swedish regional cancer registers made it possible to ascertain virtually all incident cases of renal cell cancer and the National Population Registry enabled random selection of frequency-matched population controls. In this case–control study, both cases and controls were selected from 19 counties in Sweden (covering 79% of the population) and the participation rate was relatively high.

Nevertheless, a possible limitation might be selection bias due to non-participation or non-response. Although a substantial number of cancer patients (12%) died before they could be included or were too ill to participate (6%), this would influence the results only if alcohol consumption is associated with short-term prognosis of renal cell cancer. Refusing to participate could influence the results if this was associated with alcohol consumption. Another concern is that 97% (855) of the cases but only 80% (1204) of the control subjects in the study population answered the question on alcohol consumption. This difference is mainly due to the fact that alcohol consumption was not included in the short telephone interview with the 284 control subjects who failed to answer the mailed full questionnaire. Control subjects with missing alcohol consumption had a higher BMI than those who were included in the analyses, but alcohol intake was not related to BMI in our data. Any selection bias, therefore, has probably limited influence on our findings.

We cannot rule out the possibility that misclassification of alcohol intake affected our results given that under-reporting of alcohol consumption has often been reported. However, our observed associations are not likely to be fully explained by misclassification of alcohol intake because validation studies demonstrate that self-reported alcohol assessment methods yield most realistic levels of intake if both the frequency and amount of consumption are asked for different types of alcoholic beverages separately (Feunekes *et al*, 1999). Any non-differential misclassification between cases and controls, or incorrect recalling of consumption, may lead to underestimation of the true association (Rothman and Greenland, 1998). If cases tended to under-report their intake more than controls, it would distort the observed OR towards a seemingly protective effect. However, inverse associations between alcohol intake and risk of renal cell cancer observed in prospective studies (Nicodemus *et al*, 2004; Mahabir *et al*, 2005; Rashidkhani *et al*, 2005; Lee *et al*, 2006, 2007) suggest that our results are not fully explained by such bias. Even though cigarette smoking is a risk factor for renal cell cancer (Hunt *et al*, 2005), it did not confound the associations with alcoholic beverages in our study.

The inverse association with alcohol mentioned in the above prospective studies, and in some case–control studies (Goodman *et al*, 1986; Asal *et al*, 1988; Wolk *et al*, 1996; Parker *et al*, 2002; Hu *et al*, 2003), corresponds with our results, although the findings for each specific beverage varied across studies.

A shortcoming in other studies is the inability to clearly disentangle the effect of different types of alcoholic beverage owing to limited number of only/mainly drinkers of wine, beer, or hard liquor. However, in our multivariate model with mutual adjustment for individual beverages, risk estimates did not markedly change. Because we asked for alcohol consumption 5 years before the study, we cannot account for variation in consumption over time or identify ex-drinkers in our analyses. Also, we did not ask for alcohol drinking patterns. We had insufficient information to examine associations separately by histological type of renal cell cancer.

Increased insulin sensitivity might be a mechanism by which alcohol intake reduces renal cell cancer risk. Light to moderate intake is associated with improved insulin sensitivity (Facchini *et al*, 1994; Kiechl *et al*, 1996; Lazarus *et al*, 1997; Davies *et al*, 2002) and with a lower risk of diabetes (Howard *et al*, 2004). Because obesity is a risk factor for renal cell cancer (Calle and Kaaks, 2004), and diabetics are at higher risk than those without diabetes (Wideroff *et al*, 1997; Lindblad *et al*, 1999), it is possible that improved insulin sensitivity lowers renal cell cancer risk.

A reduced risk associated with consumption of wine and beer might be due to the phenolics they contain as these possess antioxidant and antimutagenic properties (Elattar and Virji, 1999; Denke, 2000) or increase plasma antioxidant capacity in human (Ghiselli *et al*, 2000). However, the lower risk that we observed for three different alcoholic beverages and total ethanol intake suggests that alcohol itself rather than a particular type of drink is responsible for the reduction in risk. However, it is unclear why we observed an inverse association only for strong beer and not for medium–strong, or light beer, although this might be due to the lower ethanol content of light (1.8%) and medium-strong (2.8%) beer compared to strong beer (4.5%).

In conclusion, we found that moderate alcohol intake was associated with a lower risk of renal cell cancer. In particular, intake of wine, both red and white, and strong beer was associated with a reduced risk of renal cell cancer in this Swedish population.

## Figures and Tables

**Table 1:** Distribution characteristics of 855 renal cell cancer case and 1204 control subjects

	**Cases, no. (%)^a^**	**Controls, no. (%)^a^**
*Age (years)*		
20–39	18 (2)	18 (2)
40–49	58 (7)	72 (6)
50–59	185 (21)	234 (19)
60–69	263 (31)	422 (35)
70–79	331 (39)	458 (38)
		
*Body mass index (kg m^−2^)*		
⩽22.0	157 (18)	287 (24)
22.1–23.5	184 (22)	284 (24)
23.6–24.9	187 (22)	293 (24)
>25.0	299 (35)	288 (24)
		
*Cigarette smoking*		
Never smoked	381 (45)	580 (48)
⩽16.7 pack-years	240 (28)	310 (26)
>16.7 pack-years	225 (26)	280 (23)
		
*Education (years)*		
<10	539 (63)	712 (59)
10–12	177 (21)	241 (20)
>12	138 (16)	244 (20)
		
*Hypertension*		
Ever	318 (37)	291 (24)
Never	534 (62)	909 (75)
		
*Diabetes*		
Yes	102 (12)	65 (5)
No	747 (87)	1134 (94)

a Numbers and percentages do not add up to the total because of missing values for specific variables.

**Table 2:** OR and 95% CI for total ethanol intake 5 years before study and risk of renal cell cancer

			**Model 1^a^**	
	**Cases**	**Controls**	**OR**	**95% CI**
*Total ethanol* b				
Non-users alcohol	136	179	1.0	Reference
⩽54.8 g month^−1^	202	258	1.0	0.7–1.4
54.9–148.9 g month^−1^	171	255	0.9	0.7–1.3
149.0–313.6 g month^−1^	185	256	0.9	0.7–1.2
313.6–620 g month^−1^	115	163	0.9	0.6–1.3
>620 g month^−1^	46	93	0.6	0.4–0.9
*P* for trend			0.03	

BMI=body mass index; CI=confidence interval; OR=odds ratio.

a Model 1: adjusted for age, sex, BMI (quartiles), and cigarette smoking (three categories).

b All alcoholic beverages converted to grams of ethanol.

**Table 3:** OR and 95% CI for different types of alcoholic beverages 5 years before study and risk of renal cell cancer

			**Model 1^a^**		**Model 2^b^**	
	**Cases**	**Controls**	**OR**	**95% CI**	**OR**	**95% CI**
*Light beer* c						
Non-users light beer	302	377	1.0	Reference	1.0	Reference
Glasses per month⩽4	211	315	0.8	0.6–1.0	0.8	0.6–1.0
4<glasses per month⩽8	98	148	0.9	0.6–1.2	0.9	0.6–1.2
8<glasses per month⩽14	89	121	1.0	0.7–1.4	1.0	0.7–1.4
>14 glasses per month	101	130	1.1	0.8–1.5	1.1	0.8–1.6
						
*Medium-strong beer*						
Non-users medium-strong beer	437	580	1.0	Reference	1.0	Reference
Glasses per month⩽4	175	250	0.9	0.7–1.2	0.8	0.7–1.1
4<glasses per month⩽8	86	143	0.8	0.6–1.1	0.8	0.6–1.1
8<glasses per month⩽16	61	102	0.8	0.5–1.1	0.8	0.5–1.1
>16 glasses per month	96	129	1.0	0.7–1.4	1.0	0.7–1.4
						
*Strong beer*						
Non-users strong beer	620	839	1.0	Reference	1.0	Reference
Glasses per month⩽1	82	124	0.8	0.6–1.1	0.8	0.6–1.1
1<glasses per month⩽3	50	69	1.0	0.6–1.4	1.0	0.7–1.5
3<glasses per month⩽8	68	109	0.8	0.5–1.1	0.8	0.5–1.1
>8 glasses per month	35	63	0.6	0.4–1.0	0.6	0.4–1.0
						
*White wine*						
Non-users white wine	500	616	1.0	Reference	1.0	Reference
Glasses per month⩽1	116	180	0.8	0.6–1.0	0.7	0.5–0.9
1<glasses per month⩽3	84	133	0.8	0.6–1.0	0.7	0.5–1.0
3<glasses per month⩽8	111	194	0.7	0.5–0.9	0.6	0.5–0.9
>8 glasses per month	44	81	0.7	0.4–1.0	0.7	0.5–1.1
						
*Red wine*						
Non-users red wine	498	631	1.0	Reference	1.0	Reference
Glasses per month⩽1	101	163	0.8	0.6–1.0	0.7	0.5–1.0
1<glasses per month⩽4	137	188	1.0	0.7–1.3	0.9	0.7–1.2
4<glasses per month⩽8	62	106	0.8	0.5–1.1	0.7	0.5–1.1
>8 glasses per month	57	116	0.6	0.4–0.9	0.7	0.5–1.0
						
*Strong wine*						
Non-users strong wine	607	815	1.0	Reference	1.0	Reference
Glasses per month ⩽0.5	92	129	1.0	0.7–1.3	0.9	0.7–1.2
0.5<glasses per month⩽1	67	110	0.8	0.6–1.1	0.7	0.5–1.0
1<glasses per month⩽2	37	65	0.8	0.5–1.2	0.8	0.5–1.2
>2 glasses per month	52	85	0.9	0.6–1.3	0.8	0.6–1.2
						
*Hard liquor*						
Non-users hard liquor	357	487	1.0	Reference	1.0	Reference
Glasses per month⩽1	138	181	1.0	0.8–1.4	1.0	0.8–1.3
1<glasses per month⩽4	159	248	0.8	0.6–1.1	0.8	0.6–1.1
4<glasses per month⩽9	72	110	0.9	0.6–1.3	0.9	0.6–1.3
>9 glasses per month	129	178	0.9	0.7–1.3	0.9	0.7–1.4

BMI=body mass index; CI=confidence interval; OR=odds ratio.

a Model 1: adjusted for age, sex, BMI (quartiles), and cigarette smoking (three categories).

b Model 2: adjusted for age, sex, BMI (quartiles), cigarette smoking (three categories), and the other six beverages (continuous).

c Numbers do not add up to the total because of missing values.
